# Changes in plasma bile acids are associated with gallbladder stones and polyps

**DOI:** 10.1186/s12876-020-01512-8

**Published:** 2020-10-31

**Authors:** Linshi Wu, Yinping Wang, Sibo Zhu, Xunxia Bao, Zhiliang Fu, Timing Zhen, Zhiqing Yuan, Qiwei Li, Zheng Deng, Jianhua Sun, Tao Chen

**Affiliations:** 1 Department of General Surgery, South Campus, Renji Hospital, School of Medicine, Shanghai Jiaotong University, Shanghai, China; 2 Department of Biliary-Pancreatic Surgery, South Campus, Renji Hospital, School of Medicine, Shanghai Jiaotong University, No. 2000 Jiangyue Road, Pujin Street, Minhang District, Shanghai, 201100 China; 3 School of Life Sciences, Fudan University, Shanghai, 200433 China; 4 Cinoasia Institute, No.1688 North Guoquan Road, Yangpu District, Shanghai, 200438 China

**Keywords:** Bile acids, Gallbladder stones, Polyps, Gallbladder

## Abstract

**Background:**

The development of gallbladder disease (GBD) is related to bile acid (BA) metabolism, and the rate of BA circulation increases the risk of biliary cancer. However, it is unclear whether patterns of circulating bile acids (BAs) change in patients with benign GBDs such as gallbladder stones and polyps. Herein, we compared and characterised plasma BA profiles in patients with cholecystolithiasis and non-neoplastic polyps with healthy controls, and explored relationships between plasma BA profiles, demographics, and laboratory test indices.

**Methods:**

A total of 330 subjects (13 healthy controls, 292 cholecystolithiasis and 25 non-neoplastic polyps) were recruited and plasma BA profiles including 14 metabolites from patients with pathologically confirmed cholecystolithiasis and non-neoplastic polyps were compared with controls. BAs were quantitated by liquid chromatography and mass spectrometry, and statistical and regression analyses of demographics and laboratory test indices were performed.

**Results:**

Females displayed a higher burden of GBD than males (63.36% cholecystolithiasis, 60% non-neoplastic polyps). Cholecystolithiasis and non-neoplastic polyps were associated with increased plasma total secondary BAs, while levels of primary BAs were lower than in healthy controls. Plasma ursodeoxycholic acid (UDCA), tauroursodeoxycholic acid (TUDCA), glycyurdeoxycholic acid (GUDCA), taurochenodeoxycholic acid (TCDCA) and glycochenodeoxycholic acid (GCDCA) were decreased significantly in GBDs, and ursodeoxycholic acid (UDCA) was negatively correlated with white blood cell count and neutrophil percentage.

**Conclusions:**

Secondary BA levels were higher in patients with cholecystolithiasis and non-neoplastic polyps. White blood cell count and percentage of neutrophil in peripheral blood were negatively correlated with UDCA, indicating an anti-inflammation effect of UDCA.

## Background

Gallbladder disease (GBD) is a common cause of upper abdominal pain, and its incidence is significantly increased in patients with diabetes [1]. Changes in the function of gallbladder can lead to the occurrence of various diseases such as cholecystitis, gallstones, and polyps in the gallbladder.

Up to 20% of adults will develop gallstones, of which one-fifth will cause persistent pain and complications [2]. Cholecystolithiasis consists mainly of cholesterol stones or mixed stones composed primarily of cholesterol. The main underlying factor is change in the composition and properties of bile, resulting in the supersaturation of cholesterol in bile, which precipitates and crystallises readily to form stones. The formation of stones in the gallbladder can stimulate gallbladder mucosa. Gallstones cause ~ 90% of cases of acute cholecystitis and can even lead to gallbladder cancer, which can be life-threatening [3]. Laparoscopic cholecystectomy is the standard treatment for patients with symptomatic cholecystolithiasis. Gallbladder polyps are typically lesions in which the gallbladder mucosa protrudes or bulges into the gallbladder cavity, and its prevalence in adults is between 0.3 and 12.3% [4]. Polyps can be divided into neoplastic and non-neoplastic polyps in terms of pathology, and the main method of radiological diagnosis and monitoring of polyps is abdominal ultrasound.

Bile acids (BAs) have been shown to play a key role in metabolic homeostasis, and the gallbladder functions in various pathological environments, the gallbladder chelates BAs or alters BA components [5]. Chenodeoxycholic acid (CDCA) can effectively promote the dissolution of cholesterol gallstones [6], and it can be further metabolised to generate the 7β epimer ursodeoxycholic acid (UDCA) or dehydroxylated to form lithocholic acid (LCA), which can help dissolve stones.

With the advent of laparoscopic cholecystectomy, interest in the pharmacological treatment of GBD has decreased. However, cholecystectomy may increase the rate of intestinal circulation of BAs, leading to metabolic effects that increases the risk of non-alcoholic fatty liver, liver cirrhosis, and small intestinal carcinoid tumours. Therefore, it is necessary to study BAs, BA binding, and metabolic patterns in GBD and determine the relationship between these changes and disease severity.

The purpose of the present study was to compare and characterise plasma BA profiles in cholecystolithiasis and non-neoplastic polyps relative to controls with no known GBD and explore relationships between plasma BA profiles, demographics, and laboratory test indices. The results could help us understand the potential role of BAs in the occurrence and development of GBD and facilitate clinical prediction and prevention based on changes in specific indicators. The findings provide a theoretical basis for the design of oral BA drugs for the treatment of GBD.

## Methods

### Research participants

We retrospectively selected patients who meet the following criteria from existing databases (inclusion criteria: pathological diagnosis in line with surgical standards; exclusion criteria: preoperative liver dysfunction such as jaundice, common bile duct stones, pancreatitis and other diseases that may affect BAs). The time frame of patient data is November 2018 to June 2019. All participants were subjected to a medical history inquiry and physical examination, and relevant data were recorded. All participants underwent gallbladder B-mode ultrasound examination diagnosis to further distinguish cholecystolithiasis from non-neoplastic polyps according to defined criteria (cholecystolithiasis: there are one or more strong light masses in the gallbladder, accompanied by sound shadow behind, and move with the change of body position; non-neoplastic polyps: there are one or more strong light spots or strong echo protuberances on the inner wall of gallbladder, and there is no shadow behind the gallbladder, which does not move with the change of body position. The diameter of the lump is less than 1.0 cm). The control group showed no clinical, biochemical, or imaging evidence of GBD. Bile acid examination was routine examination before operation in Renji Hospital, and no additional fees are charged. Primary bile acids are synthesized directly from cholesterol by hepatocytes. Secondary bile acid is formed by the primary bile acid entering the intestine along with the bile. In the ileum and the upper part of the colon, it is catalyzed by the intestinal bacteria enzyme and converted by the debinding reaction and removing the hydroxyl group.

The study was conducted with the written informed consent of all participants, and the detailed data collection procedure obtained permission from the Ethics Committee of Renji Hospital Affiliated to Shanghai Jiaotong University School of Medicine.

### Sample collection and processing

Blood samples were collected from all patients under fasting state in early morning within 60 days after B-mode ultrasound examination. Briefly, 5 mL of blood was collected in an Ethylene Diamine Tetraacetic Acid (EDTA) anticoagulation tube and sent to the hospital laboratory for the blood test within 1 h. Plasma samples were prepared using the automated MicroLabSTAR system and placed in tubes containing EDTA for BA analysis. At room temperature, 100 μL of test sample was transferred into a 1.5 mL centrifuge tube, and 500 μL of Extract 1 (T1) was added and vortexed for 0.5 min. The sample was then centrifuged at high speed for 5 min, and 400 μL of supernatant was transferred to another centrifuge tube and dried gently.

### Liquid chromatography–mass spectrometry (LC–MS)

LC–MS was performed using a Liquid Chromatograph instrument (Shimadzu Corporation) and an API3200 mass spectrometer (American Applied Biosystems) with an electrospray ionisation (ESI) source and a linear ion trap (LIT) mass analyser. Samples were divided into two, and 100 μL of composite solution (F2) was added and vortexed for 5 min, and 11 or more injection standards at fixed concentrations were added. One aliquot was analysed using acidic positive ion optimised conditions and the other using basic negative ion optimised conditions via two independent injections using separate dedicated columns. Extracts reconstituted in acidic conditions were gradient eluted using water and methanol, both containing 0.1% formic acid, while basic extracts were eluted with water/methanol containing 6.5 mM ammonium bicarbonate. Following LC–MS runs, metabolites were identified based on the combination of chromatographic and mass spectra properties by automated comparison with metabolomic library entries of purified standards. Batch normalisation was performed using the median ratio for each metabolite in duplicate ‘anchor’ samples across runs [7].

### Bioinformatics and statistical analysis

Bioinformatics analysis was performed to generate descriptive data and frequency distributions for demographic and biochemical indicators, and group means and medians were calculated via statistical analysis (Additional file 1: Table S1). Samples or indicators were discarded when values were missing, and remaining missing values were completed using the sample algorithm in the mice (v.3.8.0) package [8]. For each index, the outlier value was calculated as five times the IQR value. Samples with outlier values were discarded. After filtering, 330 samples remained. Principal component analysis (PCA) was performed using prcomp (v.4.0.2) package [9] according to demographics, laboratory test indicators and BAs, and 95% confidence intervals and key variables were plotted. We used Kruskal–Wallis test for continuous variables of demographic and clinical data, and chi-square test for categorical variables data analysis. After evaluating the distribution and variance (Additional file 1: Tables S2 and S3), Wilcoxon test was performed for bile acid data analysis. Box diagrams were drawn using ggplot (v.3.3.0) [10].

Spearman correlation coefficients were calculated between BAs and demographic and laboratory test indicators, and pheatmap (v.1.0.12) [11] was used to draw a heatmap. Scatter plots were drawn using data adhering to *p* < 0.05 and |cor| > 0.3.

## Results

### Characteristics of the study population

As shown in Table 1, the participants of this study (n = 330, including 123 males and 207 females) contained a healthy control group (n = 13, including 6 males and 7 females), a cholecystolithiasis group (n = 292, including 107 males and 185 females) and a non-neoplastic polyps group (n = 25, including 10 males and 15 females). The average age of patients with cholecystolithiasis was 51.9 ± 13.41, and the average age of patients with non-neoplastic polyps was 46.88 ± 13.85. There were more females than males in these two GBD groups (185, 63.36%; 15, 60%). Subjects with cholecystolithiasis had a higher prevalence of metabolic complications, especially hypertension (86, 29.45%), accompanied by an increase in total bilirubin (TBIL). The incidence of diabetic complications was not significantly different between groups, but levels of aspartate aminotransferase (AST) and alanine aminotransferase (ALT) were decreased in the GBD groups.

**Table 1:** Clinical and demographic data of participants

	All	Healthy	Cholecystolithiasis	Non-neoplastic Polyps	*P* value*
Patients	330	13	292	25	
Male	123 (37.27%)	6 (46.15%)	107 (36.64%)	10 (40%)	0.753
Female	207 (62.73%)	7 (53.85%)	185 (63.36%)	15 (60%)	0.753
Age (mean ± SD)	51.87 ± 14.05	60.85 ± 22.96	51.9 ± 13.41	46.88 ± 13.85	0.035
BMI (mean ± SD)	24.27 ± 12.68	24.55 ± 4.34	24.27 ± 3.58	24.01 ± 2.95	0.787
Hypentension	93 (28.18%)	3 (23.08%)	86 (29.45%)	4 (16%)	0.327
Non-hypertension	237 (71.82%)	10 (76.92%)	206 (70.55%)	21 (84%)	0.327
Diabetes	17 (5.15%)	1 (7.69%)	16 (5.48%)	0 (0)	0.451
Non-diabetes	313 (94.85%)	12 (92.31%)	276 (94.52%)	25 (100%)	0.451
INR (mean ± SD)	0.94 ± 0.06	0.95 ± 0.08	0.94 ± 0.06	0.94 ± 0.06	0.970
AST (mean ± SD)	21.2 ± 7.08	24.48 ± 9.5	21.04 ± 7.03	21.36 ± 6.12	0.290
ALT (mean ± SD)	24.06 ± 16.19	31.11 ± 20.87	24.15 ± 16.31	19.28 ± 10.01	0.283
TBIL (mean ± SD)	12.66 ± 5.42	11.55 ± 4.74	12.75 ± 5.54	12.22 ± 4.26	0.705

^*^Kruskal–Wallis tests were used to compare continuous variables and chi-square tests were used to compare categorical variables

### PCA analysis

**Fig. 1:**
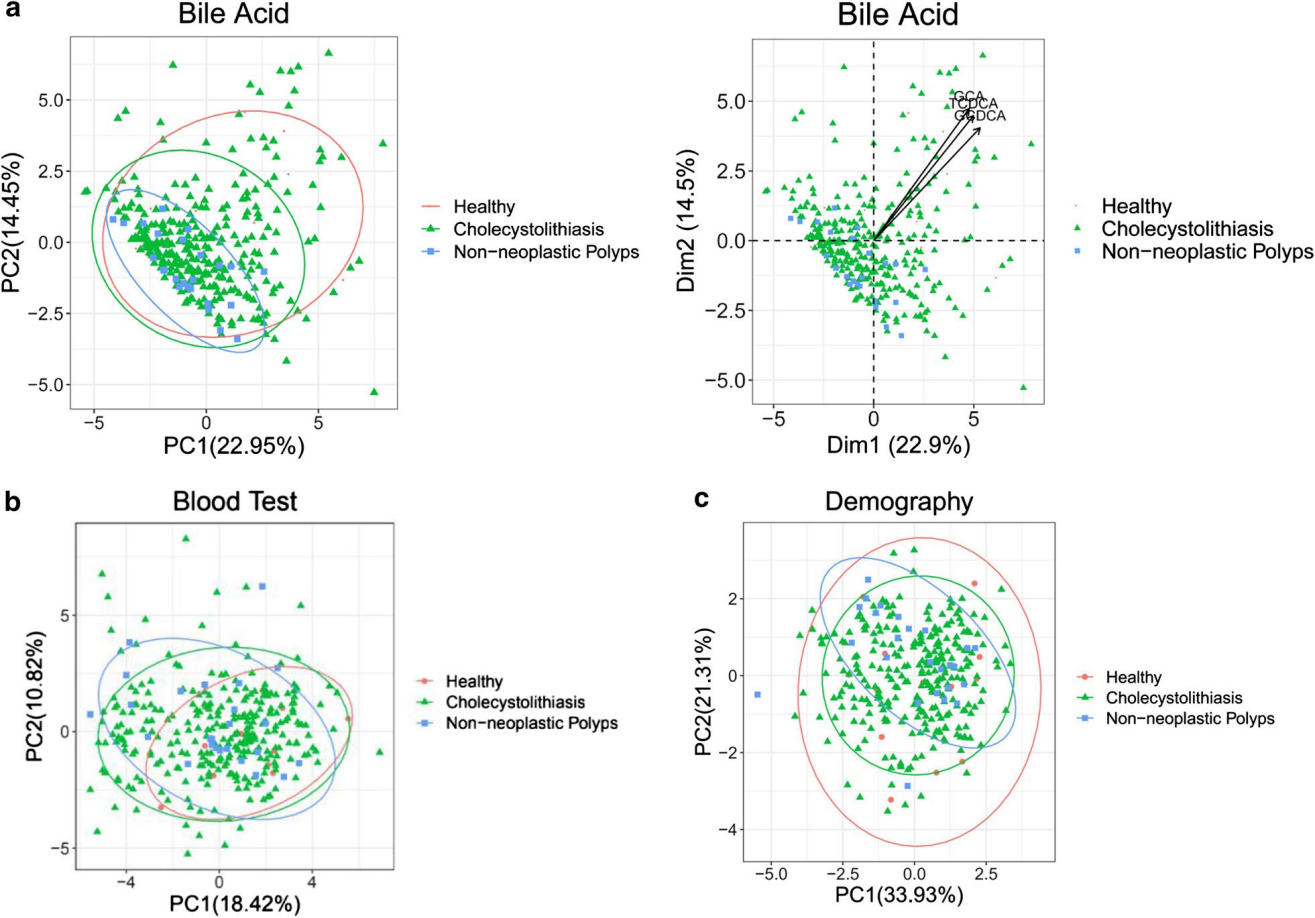
PCA cluster analysis showed that the samples of BAs in the healthy group (red) showed high PC1 and PC2 values. **a** The main reason for this difference was the change in GCA, TCDCA, and GCDCA content between patients and healthy people. **b**, **c** There is no obvious difference between blood test and demography in cluster analysis

PCA cluster analysis showed that samples in the same group were more closely clustered, and there were obvious expression differences between different groups. BAs in the healthy control group (red) yielded high PC1 and PC2 values. The main contributor to this difference was variances in GCA, taurochenodeoxycholic acid (TCDCA) and glycochenodeoxycholic acid (GCDCA) content between GDB patients and healthy controls (Fig. 1a). There was no obvious difference between groups in blood test and demography data according to cluster analysis (Fig. 1b, c).

### Plasma bile acids are altered in GDB patients

**Fig. 2:**
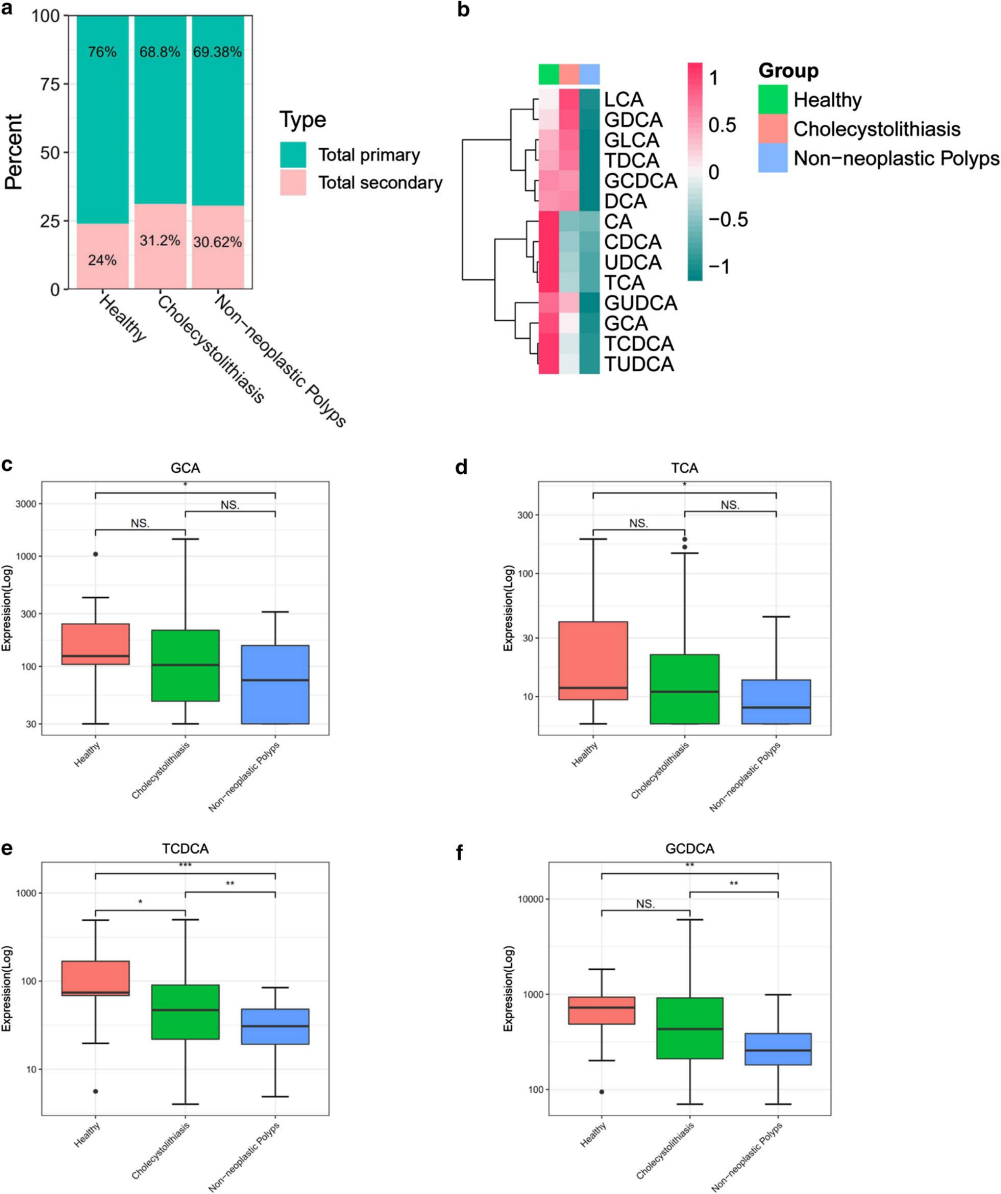
Plasma bile acid profile is significantly altered in GBD. **a** Stack bar plot representing proportion of total primary and secondary BAs. **b** Heat map display of the spectrum of BA profile across three study groups. **c**–**f** Glycine and taurine conjugates of cholate and chenodeoxycholate. *BA* bile acid; *p < 0.05; **p < 0.01; ***p < 0.001

Compared to the healthy control group, cholecystolithiasis and non-neoplastic polyp groups exhibited increase in total plasma secondary BAs. In contrast, the proportion of total primary BAs decreased (Fig. 2a). The heatmap shows differences in BAs in plasma in different groups (Fig. 2b; red = higher measured values, green = lower measured values).

### Major bile acids in plasma

Box plot comparison showed that compared to the healthy control group, patients with non-neoplastic polyps showed significant decrease in the content of glycocholate (GCA) and taurocholate (TCA)(*p* < 0.05), but there was no significant difference between the control group and cholecystolithiasis group (Fig. 2c, d). Similarly, TDCCA (*p* < 0.001) and GCDCA (*p* < 0.01) in plasma from patients with non-neoplastic polyps were also significantly reduced, and TCDCA was also significantly decreased in the cholecystolithiasis group (Fig. 2e, f).

**Fig. 3:**
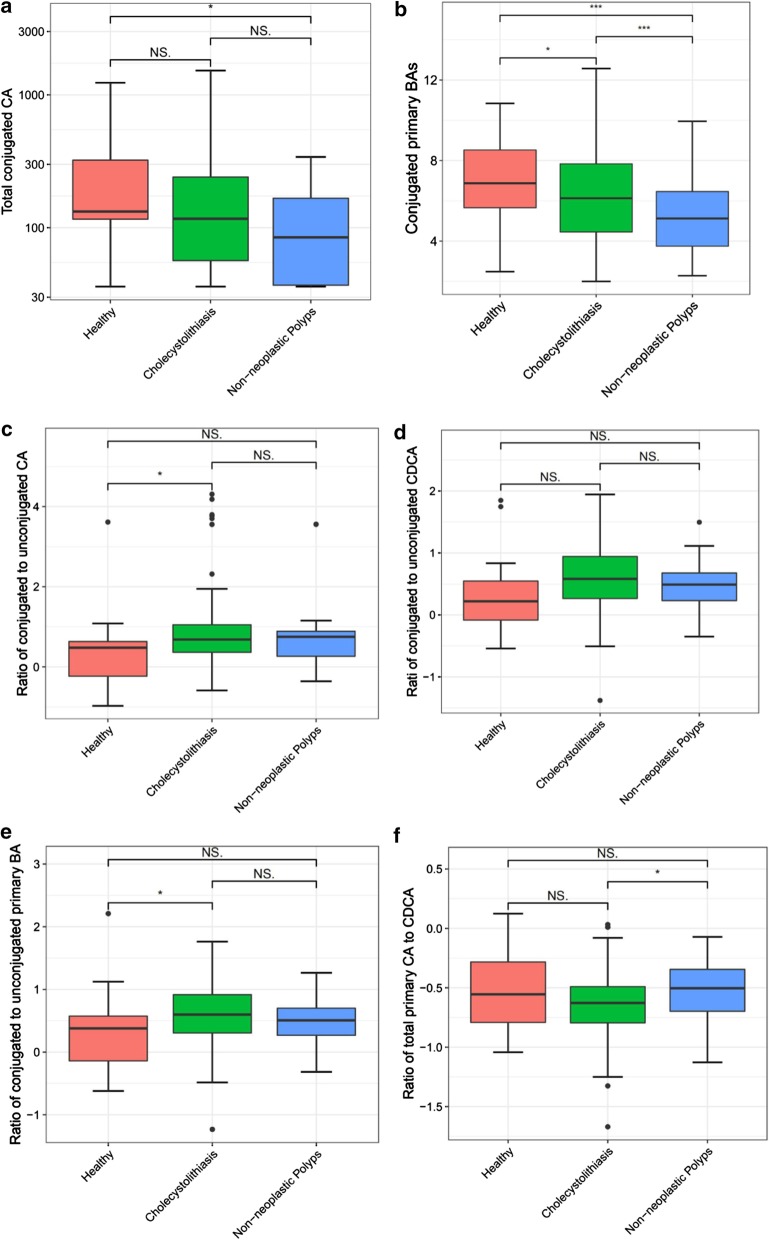
Plasma BAs along Primary BA pathway. Patients with non-neoplastic polyps demonstrate significantly decreased **a** conjugated cholate and **b** total conjugated primary BAs. Patients with cholecystolithiasis demonstrate significantly increased proportions of conjugated to unconjugated **c** cholate (CA) and **e** primary BAs. **d** cholecystolithiasis and non-neoplastic polyps did not show a significantly higher median ratio for conjugated to unconjugated CDCA. **f** Significantly increased ratio of total primary cholate (CA) to chenodeoxycholate (CDCA) in non-neoplastic polyps compared to cholecystolithiasis (the data of **cdef** graph is processed by ‘log10’ to get the ratio. x < 1, log10(x) < 0)

Total combined CA (GCA + TCA) was significantly reduced in non-neoplastic polyps patients compared with the healthy control group (*p* < 0.05), but there was no significant difference between healthy and cholecystolithiasis patients (Fig. 3a). However, total combined primary BAs (GCA + TCA + GCDCA + TCDCA) were decreased significantly in both diseases groups (Fig. 3b). Interestingly, there was an increase in the median ratio of conjugated to unconjugated CA ([GCA + TCA]/CA) in cholecystolithiasis (0.68) versus healthy controls (0.48; *p* < 0.05; Fig. 3c). Compared to healthy controls, cholecystolithiasis and non-neoplastic polyp groups did not exhibit a significantly higher median ratio of conjugated to unconjugated CDCA ([GCDCA + TCDCA]) / CDCA), as shown in Fig. 3d. Moreover, the median ratio of total conjugated to unconjugated primary BAs ([GCA + TCA + GCDCA + TCDCA]/[CA + CDCA]) was 0.38 for healthy controls, 0.60 for cholecystolithiasis patients, and 0.51 for the non-neoplastic polyp group, and difference were highly significant (*p* < 0.05) for cholecystolithiasis versus healthy control groups (Fig. 3e).

The median ratio of total cholate (CA + GCA + TCA) to total chenodeoxycholate (CDCA + GCDCA + TCDCA) was -0.55 for healthy controls, -0.63 for cholecystolithiasis patients, and -0.50 for the non-neoplastic polyp group (Fig. 3f), with a statistically significant increase in non-neoplastic polyp patients compared to cholecystolithiasis patients (*p* < 0.05).

### Plasma secondary bile acids profiles for cholecystolithiasis and non-neoplastic polyp groups

**Fig. 4:**
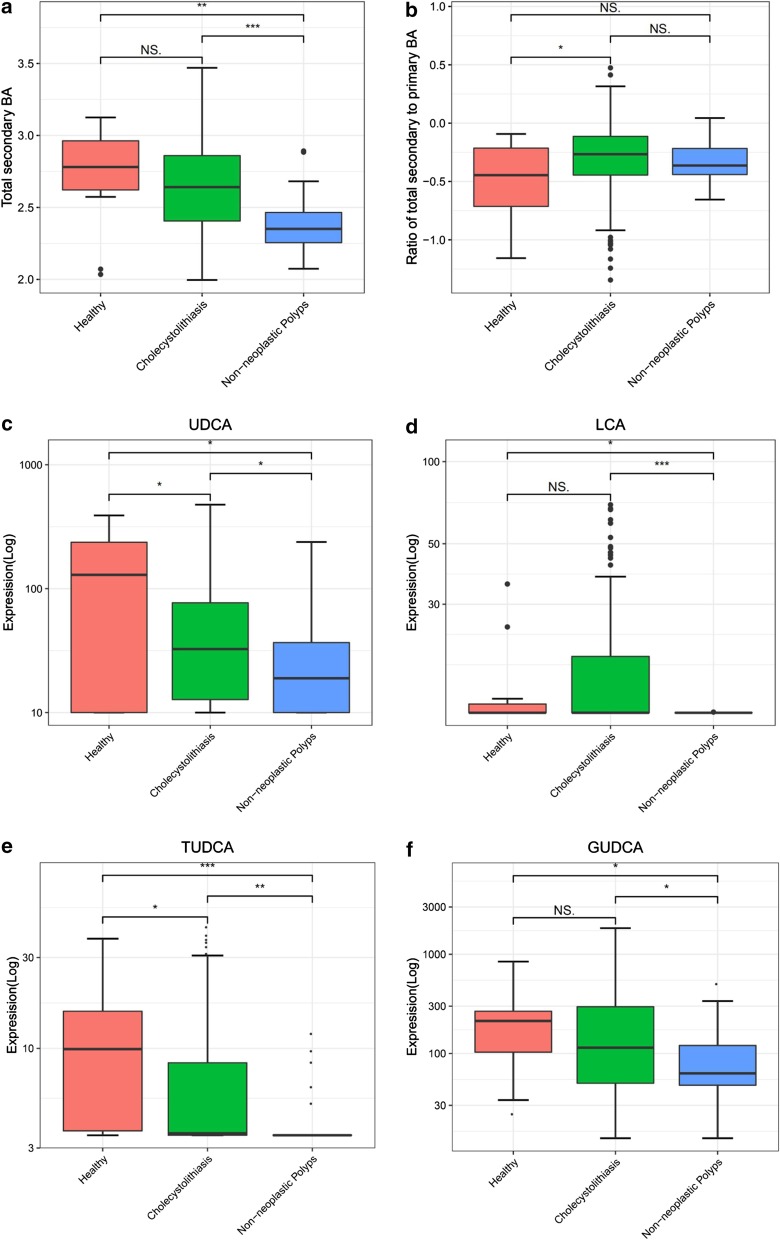
Secondary plasma bile acid changes in cholecystolithiasis and non-neoplastic polyps. **a** Secondary BA significantly decreased in non-neoplastic polyps. **b** Ratio of secondary to primary BA is significantly higher in cholecystolithiasis. **c**, **e** Subjects with non-neoplastic polyps had significantly lower ursodeoxycholate (UDCA) and tauroursodeoxycholic acid (TUDCA) versus cholecystolithiasis and healthy. **d**, **f** Lithocholate (LCA) and glycyurdeoxycholic acid (GUDCA) is significantly reduced in non-neoplastic polyps

Patients with non-neoplastic polyps had significantly lower (*p* < 0.001) total plasma secondary BAs than healthy controls (Fig. 4a). However, the total secondary to primary BA ratio was significantly higher (*p* < 0.05) in cholecystolithiasis patients relative to healthy controls (Fig. 4b). In contrast, subjects with non-neoplastic polyps had significantly lower (*p* < 0.05) UDCA and tauroursodeoxycholic acid (TUDCA) levels than cholecystolithiasis and healthy control groups (Fig. 4c, e). Similarly, LCA and glycyurdeoxycholic acid (GUDCA) were significantly reduced (*p* < 0.05) in non-neoplastic polyps (Fig. 4d, f).

### Correlation analysis between BAs and clinical features

**Fig. 5:**
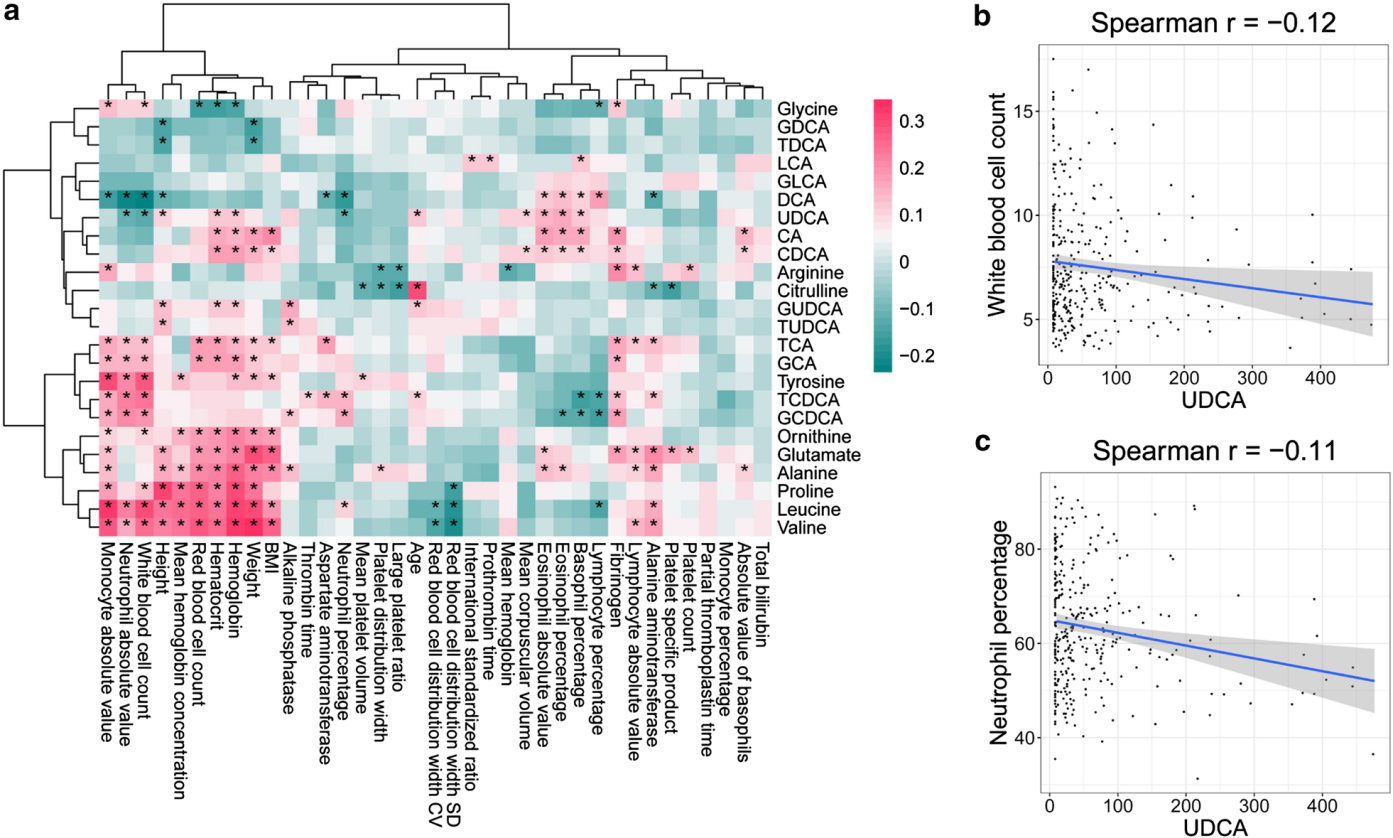
Correlation between bile acid changes and demographic indicators and blood biochemical indicators

**Fig. 6:**
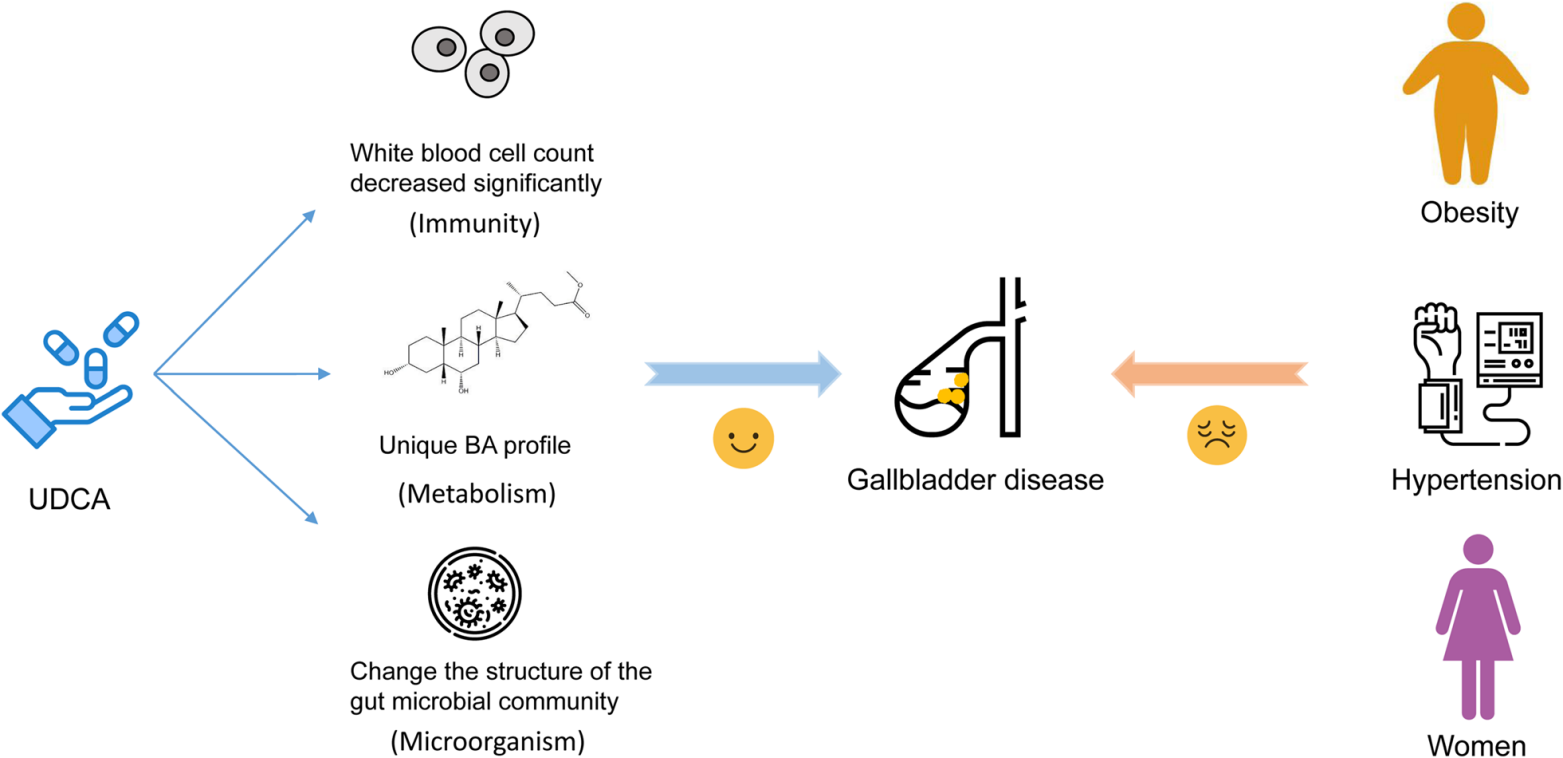
The possible mechanism of UDCA in GBD treatment. UDCA is attractive drug candidates for the treatment of GBD, since it may affect inflammatory response, unique BA profile and intestinal microbial community structure. BAs may also alter the influence of oestrogen on cholesterol metabolism, explaining why females suffer more from GDB than males. Furthermore, hypertension and obesity may serve as risk predictors of GBD. This figure was created by the author (Xunxia Bao)

Heatmaps revealed correlations between changes in BAs and demographic and blood biochemical indicators (Fig. 5a; red indicates a positive correlation, blue indicates a negative correlation, and asterisks indicate a significant correlation between the two indicators (*p* < 0.05). Meanwhile, UDCA was negatively correlated with white blood cell count and neutrophil percentage, but positively correlated with age (Fig. 5b, c). The possible mechanism of UDCA in GBD treatment is shown in Fig. 6.

## Discussion

In the present study, we acquired BA profiles for 330 subjects comprised of 13 healthy controls, 292 cholecystolithiasis patients, and 25 non-neoplastic polyp patients. Primary BAs were lower in healthy controls, and inflammation indices such as the white blood cell count and neutrophil percentage were negatively correlated with UDCA, indicating an anti-inflammation effect of UDCA.

The synthesis and excretion of BAs constitutes the main cholesterol catabolic pathway in mammals [12]. BAs are increasingly considered to be mediators and signal transduction factors in complex metabolic pathways, not just lipid solubilisers. Furthermore, the role of BAs in the occurrence and development of GBD is also receiving increasing attention. In the present study, we investigated a variety of BA abnormalities in subjects with cholecystolithiasis and non-neoplastic polyps. Correlations between plasma BA metabolome and demographic and laboratory test indices were also evaluated. Although the results cannot fully reflect cause and effect relationships, they provide a basis for the development of BA-driven GBD treatments in the future.

Plasma primary conjugated BAs were decreased significantly in both diseases, possibly due to increased intestinal and hepatic circulation leading to metabolic disorders and/or an increase in small intestinal bacteria that can dissociate primary BAs. The potential impact of intestinal and biliary flora on the pathogenesis of cholesterol gallstones cannot be ignored. Indeed, it has been reported that patients with cholecystolithiasis have significantly elevated levels of members of the phylum Proteobacteria among their gut bacteria compared with healthy people [13]. Although changes in the microbiome have been reported, the mechanism of the link underpinning these changes and the development of GBD requires further investigation.

The synthesis of BAs and cholesterol is induced in GBD, and it precedes gallstone development. This presumably occurs as a response to increased intestinal loss of BAs [14]. Few studies have investigated links between GBD and BAs, and pharmacological prevention is not currently recommended for the general population [15]. However, each pathogenic factor involved in the development of GBD can be regarded as a potential therapeutic target, especially changes in UDCA. In patients with gallbladder stones, there may be a nucleating factor in the bile that secretes a large amount of mucus glycoprotein to promote nucleation and stone formation [16]. In a double-blind placebo experiment on patients with symptomatic cholesterol gallstones, it was found that treatment with UDCA can cause gallbladder bile lipid peroxidation and decreased mucin secretion, which may alleviate improve symptoms [17]. In our current study, UDCA was significantly decreased in both cholecystolithiasis and non-neoplastic polyps, suggesting that it may have a protective effect on the gallbladder. These results are consistent with previous reports showing that UDCA can prevent gallstones [18, 19]. Notably, UDCA is positively correlated with age, but negatively correlated with white blood cell count and Neutrophil percentage. Herein, the average age of members of the healthy control group was greater than that of the two disease groups, and there may be no significant difference in body mass index (BMI) due to age interactions. Conversely, the age of disease onset decreased, suggesting that a reduction in UDCA may be one of the reasons why GBD disease patients tend to be younger. The abnormal increase in the number of white blood cells in the blood of GBD patients, especially the accumulation of neutrophils, suggests that induced inflammatory damage may lead to inflammatory gallbladder polyps. Indeed, inflammatory biomarkers are associated with postoperative prognosis of patients with gallbladder cancer. Additionally, it has been reported that an increase in the ratio of neutrophils versus lymphocytes before surgery is related to shortened overall survival of gallbladder cancer patients [20]. The specific mechanism of UDCA in reducing leukocyte content is not clear. Previous studies on inflammatory bowel disease have shown that UDCA specifically inhibits TNFα-induced IL-8 release from monocytes by inhibiting TNF receptor associated factor (TRAF2) activation [21]. Whether UDCA can inhibit pro-inflammatory cytokines in GBD in the same way need to be further confirmed.

Oral administration of UDCA produces a unique BA profile with high levels of TUDCA and GUDCA in mice treated with oral UDCA [22]. TUDCA and GUDCA have been shown to have anti-inflammatory effects [23]. Hydrophilic UDCA and its taurine-conjugated form TUDCA exert strong cytoprotective effects [24]. In our current study, we also found that TUDCA and GUDCA were decreased significantly in patients, which further highlights the key role of UDCA in maintaining BA circulation homeostasis in vivo. PCA cluster analysis showed that GCA, TCDCA and GCDCA play a key role in the health of the biliary system. Additionally, our results also show that TDCCA and GCDCA tend to be lower in plasma from patients with both diseases, and the decline in TCDCA is particularly significant. The change in GCDCA is particularly pronounced in non-neoplastic polyps. Levels of these conjugated BAs are of great value for the identification of cholesterol polyps and adenomatous polyps [25].

Our study may be limited in several ways. First, our study was a retrospective, small-sized study. The unbalanced sample size of the three groups may lead to bias of the analysis results. Second, the use of single factor analysis may lead to the interference of confounders. Finally, in the selection of normal control group, the difference of age may lead to the bias of results.

## Conclusions

In summary, we demonstrated significant changes in circulating BA composition in cholecystolithiasis and non-neoplastic polyps, especially changes in UDCA, TUDCA, GUDCA, TCDCA and GCDCA. The inflammatory response caused by the interaction between UDCA and the number of white blood cells is particularly worthy of attention, as is the resulting unique BA profile, which may contribute to halt the development of GBD. BAs are attractive drug candidates for the treatment of GBD, since they may affect the intestinal microbial community structure. BAs may also alter the influence of oestrogen on cholesterol metabolism, explaining why females suffer more from GDB than males. Furthermore, hypertension and obesity may serve as risk predictors of GBD. The imbalance in the number of subjects in each group is an issue in the present work that should be addressed in future studies. While our findings are correlative, they provide a theoretical basis for the development of oral BA drugs to treat GBD.

## Supplementary information

**Additional file 1**. All statistical indicators and dunn test.

## Data Availability

The datasets used and/or analysed during the current study are available from the corresponding author on reasonable request.

## Abbreviations

AFU: Fucosidase
A/G: ALB/GLB
ALB: Albumin
ALT: Alanine aminotransferase
AST: Aspartate aminotransferase
BA(s): Bile acid(s)
BMI: Body mass index
CA: Cholic acid
CDCA: Chenodeoxycholic acid
CG: Cholyglycine
CREA: Creatinine
DBIL: Direct bilirubin
EDTA: Ethylene diamine tetraacetic acid
ESI: Electrospray ionisation
GBD: Gallbladder disease
GCA: Glycocholate
GCDCA: Glycochenodeoxycholic acid
GGT: Glutamyltranspeptidase
GLB: Globulin
GLCA: Glycosylcholic acid
GUDCA: Glycyurdeoxycholic acid
INR: International normalized ratio
LCA: Lithocholic acid
LC–MS: Liquid chromatography–mass spectrometry
LDH: Lactic dehydrogenase
LIT: Linear ion trap
PA: Prealbumin
PCA: Principal component analysis
TBA: Total bile acid
TBIL: Total bilirubin
TCA: Taurocholate
TCDCA: Taurochenodeoxycholic acid
TLCA: Taurolithocholic acid
TP: Total protein
TUDCA: Tauroursodeoxycholic acid
UDCA: Ursodeoxycholic acid
URIC: Uric acid
