# Supplementary material for: Changes in plasma bile acids are associated with gallbladder stones and polyps
Source: BMC Gastroenterol. 2020 Oct 31;20:363. doi: 10.1186/s12876-020-01512-8 (PMC7603702; doi:10.1186/s12876-020-01512-8)
Supplement: Supplementary file 1 — Additional file 1. All statistical indicators and dunn test. [file 12876_2020_1512_MOESM1_ESM.docx]

Table S1. All statistical indicators

| Classification | Index |
| --- | --- |
| Demography | Gender; Age; Height; Weight; Hypentension; Diabetes; BMI |
| Laboratory test index | INR; Prothrombin time; Thrombin time; Fibrinogen; Partial thromboplastin time; Absolute value of basophils; Eosinophil absolute value; Monocyte absolute value; Hematocrit; Basophil percentage; Mean platelet volume; Platelet distribution width; Red blood cell distribution width CV; Hemoglobin; Lymphocyte absolute value; Platelet specific product; Eosinophil percentage; Platelet count; Large platelet ratio; Mean hemoglobin; Neutrophil absolute value; Mean hemoglobin concentration; Lymphocyte percentage; Red blood cell distribution width SD; Red blood cell count; White blood cell count; Neutrophil percentage; Monocyte percentage; Mean corpuscular volume; CG; A/G; AST; ALT; AFU; LDH; DBIL; GLDH; URIC; PA ; GLB; GGT; ALB; TBIL; TBA; ALP; CREA; TP |
| Bile acids | LCA; TLCA; GLCA; UDCA; CA; Glutamate; CDCA; Ornithine; TCDCA; GCDCA; DCA; Leucine; Proline; Valine; GDCA; GUDCA; TDCA; Alanine; Citrulline; Glycine; TCA; GCA; TUDCA; Tyrosine; Homocysteine; Arginine |

Table S2. Distribution and variance for bile acids data

| Bile acids | Healthy | Cholecystolithiasis | Non-neoplastic Polyps | levene |
| --- | --- | --- | --- | --- |
| LCA | 1.00E-05 | 4.86E-25 | 1.21E-10 | 6.00E-06 |
| UDCA | 0.217241564 | 2.33E-24 | 2.85E-07 | 0.008458122 |
| CA | 0.000318724 | 1.79E-22 | 8.24E-05 | 2.05E-32 |
| CDCA | 0.001578675 | 1.01E-23 | 1.80E-06 | 2.15E-09 |
| TCDCA | 0.001857518 | 1.95E-20 | 0.148933159 | 0.000259627 |
| GCDCA | 0.176257981 | 7.92E-22 | 0.001565055 | 0.001336579 |
| DCA | 0.001972579 | 1.55E-24 | 9.93E-06 | 0.025464282 |
| GDCA | 0.055969968 | 1.97E-24 | 8.89E-05 | 0.004388423 |
| GUDCA | 0.002707294 | 6.54E-23 | 1.03E-05 | 0.006362923 |
| TDCA | 0.008769517 | 7.17E-24 | 1.56E-05 | 0.030153247 |
| TCA | 0.000502881 | 2.77E-25 | 6.10E-06 | 7.20E-05 |
| GCA | 0.000270914 | 1.85E-23 | 0.000989214 | 0.084750342 |
| TUDCA | 0.015699336 | 1.24E-24 | 3.90E-08 | 0.00068464 |

Table S3. Dunn test(as post-hoc analysis) on the continuous variable data of Table 1

|  | Cholecystolithiasis - Healthy | Cholecystolithiasis - Non-neoplastic Polyps | Healthy - Non-neoplastic Polyps |
| --- | --- | --- | --- |
| Age | 0.056390278 | 0.037676666 | 0.015757985 |
| BMI | 0.455455722 | 0.749627181 | 0.460675292 |
| INR | 1 | 0.656665581 | 0.471833759 |
| ALT | 0.140068014 | 0.203271548 | 0.175993661 |
| AST | 0.224114278 | 0.237983352 | 0.335147178 |
| TBIL | 0.327954697 | 0.395855841 | 0.631326881 |
